# Muscle elasticity is different in individuals with diastasis recti abdominis than healthy volunteers

**DOI:** 10.1186/s13244-021-01021-6

**Published:** 2021-06-29

**Authors:** Kai He, Xiuling Zhou, Yulan Zhu, Bo Wang, Xiaojian Fu, Qiyuan Yao, Hao Chen, Xiaohong Wang

**Affiliations:** 1 Department of General Surgery, Huashan Hospital, Fudan University, 12 Wulumuqi Road, Shanghai, 200040 China; 2 Department of Ultrasound, Huashan Hospital, Fudan University, 12 Wulumuqi Road, Shanghai, 200040 China; 3 Department of Rehabilitation, Huashan Hospital, Fudan University, 12 Wulumuqi Road, Shanghai, 200040 China

**Keywords:** Sonoelastography, Shear wave speed, Diastasis recti abdominis, Linea alba, Abdominal wall muscle

## Abstract

**Objective:**

To determine the value of shear wave elastography (SWE) in assessing abdominal wall muscles, including rectus abdominis (RA), external oblique muscle (EO), internal oblique muscle, and transversus abdominis (TrA) in patients with diastasis recti abdominis (DRA) and healthy controls.

**Methods:**

From October 2018 to December 2019, 36 postpartum DRA patients and 24 nulliparous healthy women were identified. Inter-rectus distance (IRD) measurements were taken by B-mode ultrasound. Shear wave speed (SWS) values were acquired by one operator at ten specific locations. Clinical and ultrasound variables, including demographics, IRD, muscle thickness, and muscle SWS, were compared between the two groups using Student’s *t* test or *Fisher's* exact test. Pearson correlation analyses were conducted for the variables of IRD, muscle thickness, and SWS in the 36 DRA patients.

**Results:**

The maximum diameter of recti abdominus separation was located at the umbilicus in DRA patients (4.59 ± 1.14 cm). The SWS value was significantly lower in the RA (*p* = 0.003) and higher in the TrA muscle (*p* < 0.001) in DRA patients compared with the age-matched controls. However, SWS in both muscles (RA and TrA) showed a statistically positive correlation with IRD (*p* < 0.05). In addition, the SWS value in EO statistically decreased in DRA patients compared with the healthy controls (1.65 ± 0.15 vs. 1.79 ± 0.14, *p* = 0.001).

**Conclusions:**

The application of SWE to abdominal wall muscles in DRA patients is feasible. The correlation between SWS value and IRD in RA should be interpreted with caution.

## Key points

- Imaging assessment using shear wave elastography of abdominal muscle elasticity.
- Abdominal muscle elasticity in DRA patients was different from healthy volunteers.
- Abdominal muscle elasticity in DRA patients was correlated with inter-rectus distance.

## Background

Diastasis recti abdominis (DRA) is the enlargement of the distance between the edges of the recti abdominis along the linea alba with no fascial defect [1, 2]. Normally, the rectus muscles are fused at the midline with no more than 1 to 2 cm separation [3]. A significant number of women are affected by DRA during the prenatal and postnatal periods [4], resulting in functional problems, such as back pain and hernia [5].

Clinically, DRA is evaluated by finger breadth. The widths of the two recti abdominis muscles are assessed by palpation 4.5 cm above and 4.5 cm below the umbilicus, along the linea alba [6]. Separation is observed most commonly at the umbilicus. A width greater than or equal to the width of two fingers is defined as DRA [5]. No separation or a width of less than two fingers is defined as no DRA. However, in the past twenty years, the reliability of palpation has been debated [1, 3, 4]. Therefore, prior studies investigated the application of advanced imaging technology, like B-mode ultrasound (US), on the evaluation of inter-rectus distance (IRD) measurement. Most studies demonstrate the superior accuracy and validity of imaging tools compared to palpation [7–9].

Clinical management of DRA is challenging [10]. Based on the pathophysiology described by Baumann et al. [11], the combination of physiotherapy and surgical repair has the potential to improve the anatomical divarication and the laxity of the ventral abdominal muscles. In other words, the preoperative assessment of DRA is not solely a distance measurement problem. Individual preoperative evaluation of physiologic tension is important. Up to now, conventional modalities, including B-mode ultrasound, computed tomography, and magnetic resonance imaging, have been used to assess the IRD, but no functional biomechanical property evaluation was available. Therefore, an objective imaging assessment of the intrinsic abdominal muscle characteristics, such as the elasticity, is needed to predict successful treatment and disease progression.

In recent years, ultrasound shear wave elastography (SWE) has been increasingly used to measure physiologic and pathologic muscle behavior due to its non-invasiveness, high accuracy, user-friendliness, and availability in commercial ultrasound scanners [12–14]. However, to this end, no convincing evidence in the current literature has established the application of SWE for detecting muscle properties in DRA patients. Based on our previous study of SWE in incisional hernia patients [15], we hypothesize that the elasticity in rectus abdominis (RA) of DRA patients is significantly lower than that in healthy controls, while the elasticity of lateral abdominal wall muscles, including the external oblique (EO) muscle, internal oblique (IO) muscle, or transversus abdominis (TrA), is comparatively higher.

This study aimed to determine the utility of applying SWE to the evaluation of abdominal wall muscles, including rectus abdominis, external oblique muscle, internal oblique muscle, and transversus abdominis, in patients with DRA and healthy controls, to assist in clinical management. No study has been reported on the SWE of abdominal wall muscles in DRA patients.

## Methods

### Patients

This study was reviewed and approved by the Institute Ethics Committee of Huashan Hospital (No. KY2018-438). Informed consent forms were signed by all participants. The study protocol was registered in China Clinical Registry Center (No. ChiCTR1900023012). From October 2018 to December 2019, postpartum women suspected of DRA at the outpatient clinic in our institution were identified. Eligible subjects were recruited based on the following inclusion criteria: 1) age between 18 and 60 y/o; 2) IRD greater than or equal to 2-finger width on palpation, irrespective of the locations along the midline, measured in the standard supine crock-lying position with arms crossed over the chest; and 3) full-term fetus. The exclusion criteria for all postpartum women were as follows: (1) additional history of abdominal surgery or injury (except cesarean section); (2) chronic or degenerative pathology of the muscle (e.g., autoimmune myositis); (3) abdominal rehabilitation of neuromuscular electrical stimulation within the previous 6 months; and (4) inadequate clinical and ultrasound (US) imaging data. Nulliparous age-matched women were recruited from the physicians and nurses at our institution to participate as healthy controls. The exclusion criteria for the healthy participants were similar to that for DRA patients, including age (< 18 and > 60 y/o), surgery, and history. The clinical variables of age, weight, and height were recorded for each subject.

### US data collection

#### Equipment

Ultrasound images were obtained using a high-end scanner (Aixplorer, Supersonic Imagine, France) equipped with an SWE mode (general preset). The scanner was coupled with a linear array probe (SL10-2, Supersonic Imagine, France). Elastography was performed to evaluate tissue elasticity. Elasticity is the tendency of tissue to resist deformation against an applied force or to resume its original shape after removal of this force. A higher elastic modulus correlates with a higher resistance to deformation and an increased stiffness [16]. Shear wave speed is a quantitative measure of tissue stiffness and can be converted to shear modulus using the following equation:

$$\mu=C_s^2\rho$$

where *µ* is shear modulus; *C*_*s*_ is shear wave speed (SWS) in this equation; and *ρ* is density, which can be assumed to be 1000 kg/m^3^ for all soft tissues [17]. Higher speed values are associated with increased tissue stiffness. A senior radiologist (XW.: with 10 years of experience in abdominal and musculoskeletal ultrasound imaging) performed the IRD measurement on B-mode US and SWS measurement on SWE.

#### IRD measurement

The DRA patients underwent B-mode US examination of their anterior rectus abdominis sheath to evaluate the width of rectus diastasis. To standardize the position of the transducer, each measurement location was marked on the skin when the participant was resting in the supine position, with their arms across their chests. Additionally, special attention was paid to the pressure imposed on the probe to avoid reflexive responses from the participants. A thick gel layer was applied to replace the air gap between the US transducer and the targeted region.

**Fig. 1:**
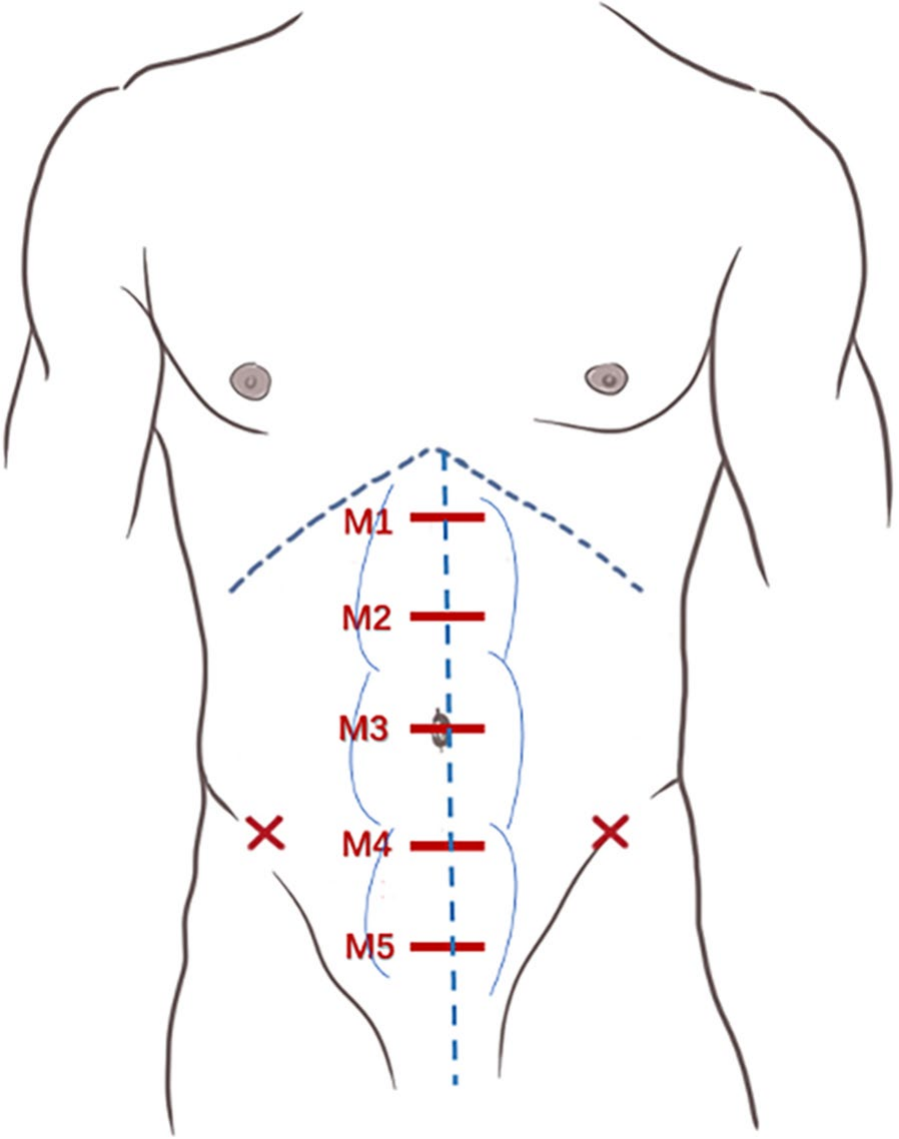
Inter-rectus distance measurement at the following five locations along the midline of the abdomen: subxiphoidal, epigastric, umbilical, infraumbilical, and suprapubic location

**Fig. 2:**
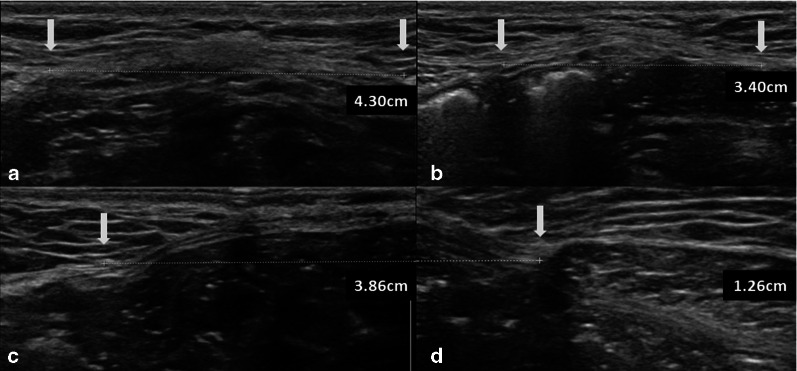
Forty-year-old woman with one previous pregnancy. Diastasis (calipers) is present at the level of M2 (above the umbilicus, **a** 4.30 cm), at the level of M4 (below the umbilicus, **b** 3.40 cm). At the level of M2 (umbilicus), measurements were combined with two separate calipers (**c** 3.86 cm, **d** 1.26 cm). White arrows pointed to the locations of calipers

According to the European Hernia Society (EHS) classification of midline incisional hernias [3], the transducer was placed transversely along the midline at five specific locations identified with skin markers in the following order: subxiphoidal, epigastric, umbilical, infraumbilical, and suprapubic location. The aforementioned locations were recorded as M1, M2, M3, M4, M5, respectively (Fig. 1). Using the medial margins of both rectus abdominis muscles, the inter-rectus distance could be clearly identified, from one side of the anterior recti sheath to the corresponding position of its counterpart on the other side. Measurements were taken with an on-screen caliper to the nearest 0.1 cm (Fig. 2). Finally, the maximum diastasis was recorded as the width of the recti abdominus separation in this study.

Further assessment of anatomical variations of rectus abdominis muscle diastasis by B-mode US was made based on a recent study by Corvino et al. [18], including the following five patterns: open only above the navel, open only below the navel, open at the navel level, open completely but wider above the navel, and open completely but wider below the navel.

#### Shear wave elastography measurement

**Fig. 3:**
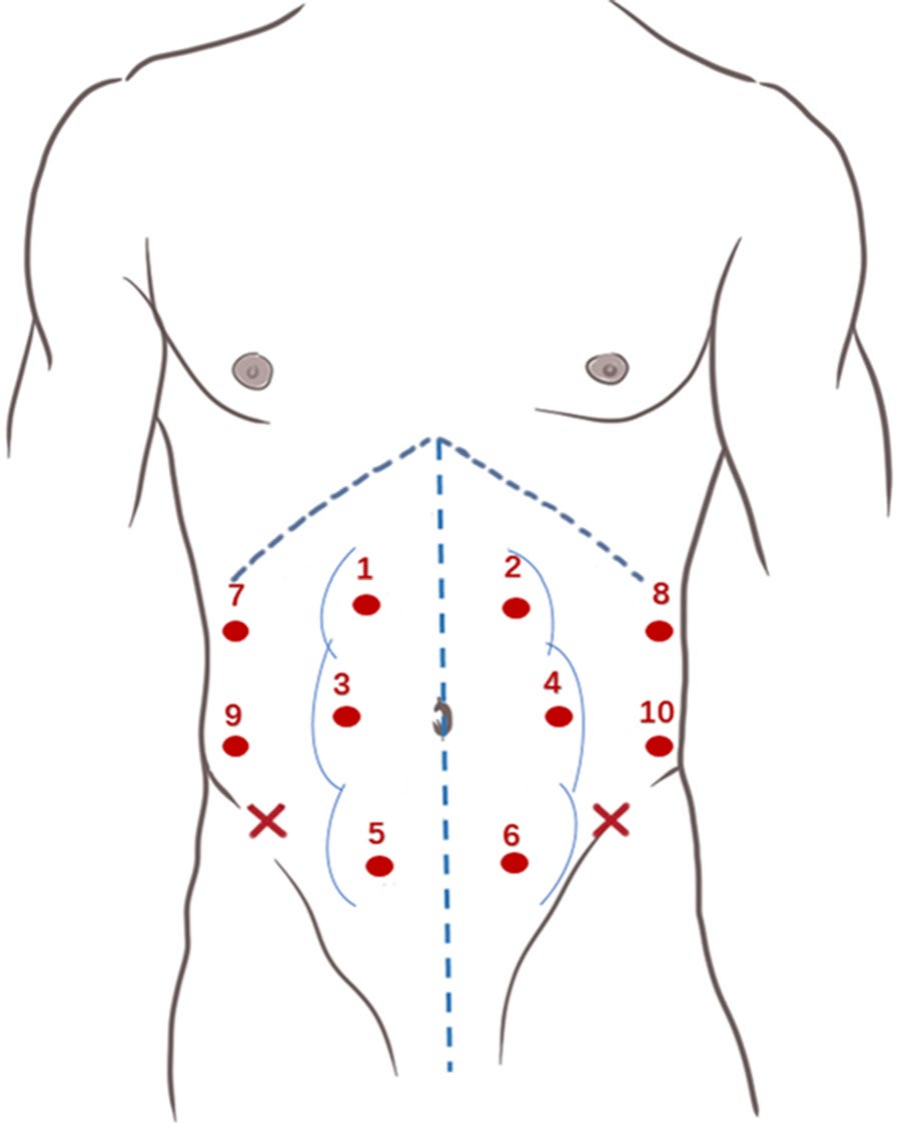
Ten specific locations for shear wave elastography measurements

After the IRD measurements, SWE was measured in the DRA patients and healthy nulliparous participants. Tissue SWS of the RA and lateral muscles (EO, IO, and TrA) were bilaterally measured. Based on our previous experience [15] and the suggestions of Rath et a. [19], SWE measurements were taken on both the left and right sides of the abdomen as per the following guidelines (Fig. 3):

1. Three locations were identified on each side of the RA region, the supraumbilicus (4.5 cm above the umbilicus), umbilicus, and subumbilicus (4.5 cm below the umbilicus).
2. Two locations were identified on each side of the abdomen along the anterior axillary line equidistant between the costal margin at the level of the ninth rib and a point anterior to the anterior superior iliac spine.

Each measurement was initiated with a B-mode acquisition of the muscle. The transducer was placed with light pressure to obtain the transverse view of the target muscle. To maximize intra-operator reliability and minimize the duration of transducer repositioning at the same location in each subject, 10 waterproof skin landmarks were drawn with a marker under 2D-mode monitoring by the same operator before SWE. The depth was set to optimize the target muscle centrally. Muscle thickness was obtained by measuring the length between the anterior and posterior layers of the sheath or myofascial.

**Fig. 4:**
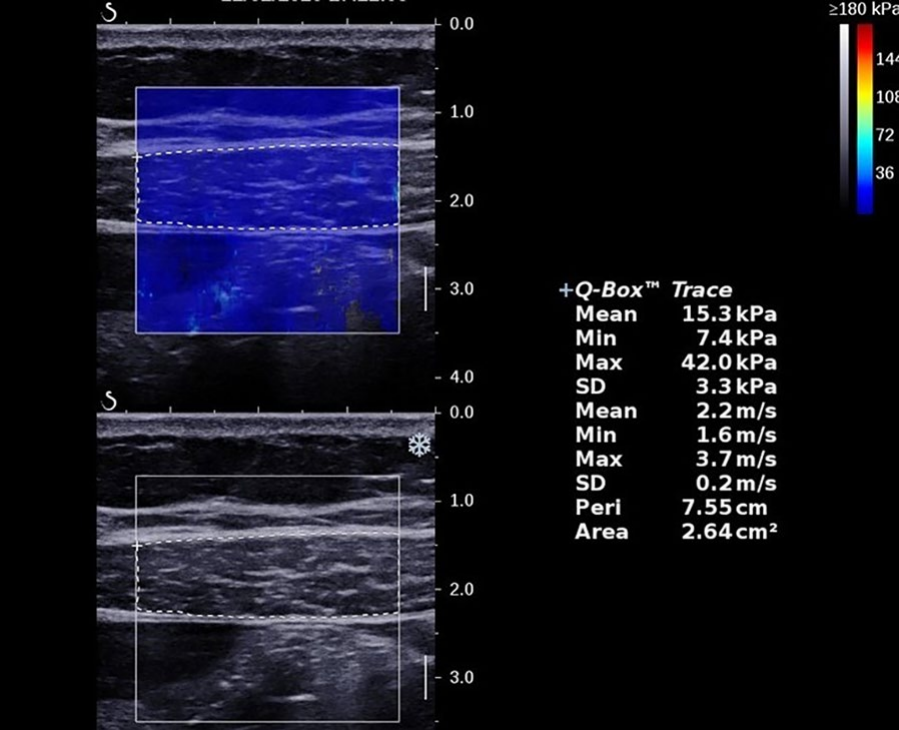
Ultrasound images of shear wave speed measurement in upper right rectus abdominis. A new region of interest (dotted line) was drawn to obtain the parameter of mean shear wave speed

After landmarks were identified, the SWE mode was turned on. The rectangle-shaped SWE box was centrally placed to contain the target muscle, avoiding the tendons, aponeurosis, blood vessels, and fascial tissues. Laterally, the size of the SWE box was set to the maximum diameter. The anterior–posterior diameter of the SWE box was adjusted to contain the whole muscle in the transverse view. A static SWE image was acquired and stored when the real-time color map was as homogeneous as possible for at least 5 s. The operator then manually drew a new region of interest ROI based on the anatomic contour of the muscle. The shear modulus (*μ*) and shear wave speed (*C*_*s*_) values were both automatically reported by the scanner. In the current study, the mean shear wave speed was adopted as the outcome measure, based on prior experience [15] (Fig. 4). After SWE measurements were taken at ten specific locations, additional two SWE sessions were performed to assess the intra-operator reliability. Consequently, the mean SWS value of the three sessions represented the final SWS value of the muscle.

### Statistical analysis

The results are presented as mean ± standard deviation for the numerical variables of muscle thickness and shear wave speed. The Student’s *t* test and Fisher's exact test were adopted to compare the clinical variables, B-mode, and SWE parameters in DRA patients and healthy volunteers. A *p* value < 0.05 indicated a significant difference. The intra-operator reliability was calculated using the intra-class correlation coefficient (ICC). The ICC was interpreted as follows: 1.00–0.75, excellent; 0.74–0.60, moderate; 0.59–0.40, fair; and < 0.40, poor [20]. The Pearson correlation coefficient was calculated to assess the correlation between IRD, muscle thickness, and SWS. The DRA patients were divided into three subgroups based on their IRD. SWS values were compared among the subgroups using one-way ANOVA. Statistical analyses were performed using SPSS for Windows version 21 (SPSS Institute, Cary, NC, USA).

## Results

### Patients summary

**Fig. 5:**
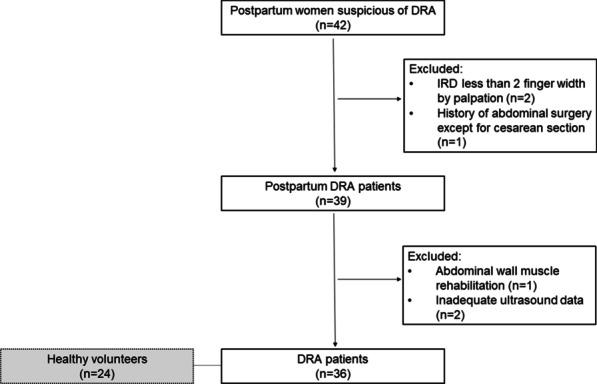
Patient recruitment flowchart

A power analysis was performed to determine the number of patients needed. We assumed that the mean value for mean trunk rotation torque was 37 (N·m) in DRA patients and 45.3 (N·m) in non-DRA patients, based on the data reported by Hills et al. [21]. The probability was 80 percent that the study would detect a difference of 8.3, based on a standard deviation of 3.6, the mean trunk rotation torque, and a two-sided 0.05 significance level. Thus, 44 subjects (22 patients and 22 controls) were required for this retrospective study. Accordingly, 24 nulliparous healthy women were recruited for this study. From October 2018 to December 2019, 42 postpartum women suspected of DRA at our outpatient clinic were identified. After removing 6 patients, 36 patients were enrolled for the final analysis. Among the excluded patients were (a) 2 patients with IRD less than 2-finger width, (b) 1 patient with additional surgical history except for cesarean section, (c) 1 patient with recent abdominal rehabilitation of neuromuscular electrical stimulation, and (d) 2 patients without adequate ultrasound data (Fig. 5).

### Intra-operator reliability analyses of shear wave speed

A total of 3240 SWS values were acquired by one operator, including three measurements per location for sixty subjects. Interclass correlation analyses showed good moderate to excellent intra-operator reliability in terms of each abdominal wall muscle (Table 1), with coefficients ranging from 0.681 to 0.851.

**Table 1:** Intra-operator reliability analyses of shear wave speed measurement

Findings	ICC	95%CI
Session 1 versus Session 2		
RA	0.851	0.708, 0.924
EO	0.681	0.374, 0.837
IO	0.772	0.554, 0.884
TrA	0.813	0.633, 0.905
Session 1 versus Session 3		
RA	0.849	0.704, 0.923
EO	0.762	0.538, 0.878
IO	0.713	0.437, 0.854
TrA	0.879	0.762, 0.938

*ICC* interclass correlation coefficient

### Demographics and elastographic results

The clinical and sonographic features of the 60 subjects (DRA = 36, non-DRA = 24) are summarized in Table 2. No statistically significant differences were found in the age, height, and BMI between the two groups (*p* > 0.05). Among the DRA patients, 94.4% (34/36) had lumbopelvic pain. IRD measurements revealed that the maximum diameter of recti abdominus separation was located at the umbilicus (M3) in the DRA patients (4.59 ± 1.14 cm). The rectus muscles were fused at the midline without separating in the 24 healthy participants. The RA, EO, and TrA in DRA patient groups were significantly thinner than those in the control group (7.97 ± 1.60 vs. 9.99 ± 1.33 mm, *p* < 0.001; 5.11 ± 1.05 vs. 6.18 ± 1.07 mm, *p* < 0.001; 2.42 ± 0.45 vs. 2.96 ± 0.78 mm, *p* = 0.004, respectively). In addition, SWS propagated significantly slower in the RA and EO of DRA patients (*p* = 0.003, *p* = 0.001, respectively). Conversely, the SWS propagated significantly faster in the TrA of DRA patients (*p* < 0.001).

**Table 2:** Frequency of clinical and US features in 36 DRA patients and 24 nulliparous healthy controls

Findings	DRA (*n* = 36)	Non-DRA (*n* = 24)	Total (*n* = 60)	*p* value
Age (y) *	28.56 ± 3.70	26.71 ± 4.71	27.82 ± 4.20	0.095
Height (cm)	161.57 ± 4.90	163.08 ± 4.84	162.18 ± 4.89	0.243
BMI prepregnancy (kg/m^2^)	20.17 ± 2.00	20.59 ± 2.15	20.34 ± 2.06	0.438
BMI predelivery (kg/m^2^)	25.97 ± 3.03	/	/	/
Weight gain (kg)	15.09 ± 5.51	/	/	/
Birth weight (kg)	3.71 ± 0.68	/	/	/
Delivery mode				
CS	32	/	/	/
VD	4	/	/	/
Lumbopelvic pain				
Yes	34	0	34	< 0.001
No	2	24	26	
IRD (cm)				
M1	0.93 ± 0.81	0	0.49 ± 0.75	< 0.001
M2	3.00 ± 1.28	0	1.80 ± 1.78	< 0.001
M3	4.59 ± 1.14	0	2.76 ± 2.43	< 0.001
M4	2.16 ± 1.35	0	1.30 ± 1.49	< 0.001
M5	0.19 ± 0.49	0	0.10 ± 0.36	0.052
DRA pattern		/	/	/
Pattern 1 (only above navel)	5	/	/	/
Pattern 2 (only below navel)	0	/	/	/
Pattern 3 (at navel level)	0	/	/	/
Pattern 4 (complete but wider above navel)	26	/	/	/
Pattern 5 (complete but wider below navel)	5	/	/	/
Muscle thickness (mm)				
RA	7.97 ± 1.60	9.99 ± 1.33	8.80 ± 1.79	< 0.001
EO	5.11 ± 1.05	6.18 ± 1.07	5.54 ± 1.17	< 0.001
IO	5.95 ± 1.09	5.49 ± 1.22	5.76 ± 1.16	0.135
TrA	2.42 ± 0.45	2.96 ± 0.78	2.63 ± 0.66	0.004
SWS (m/s)				
RA	1.69 ± 0.20	1.82 ± 0.13	1.74 ± 0.18	0.003
EO	1.65 ± 0.15	1.79 ± 0.14	1.71 ± 0.16	0.001
IO	1.62 ± 0.15	1.54 ± 0.15	1.59 ± 0.16	0.070
TrA	1.68 ± 0.22	1.45 ± 0.18	1.59 ± 0.23	< 0.001

*DRA* diastasis recti abdominis, *BMI* body mass index, *CS* cesarean section, *VD* vaginal delivery, *IRD* inter-rectus distance, *RA* rectus abdominis, *EO* external oblique, *IO* internal oblique, *TrA* transversus abdominis, *SWS* shear wave speed

As shown in Table 3, the SWS values for the RA, EO, IO, and TrA varied at different locations between the groups. Specifically, in the DRA patients, the SWS showed significantly lower SWS value in the upper RA and at the umbilicus (locations of 1, 2, 3, and 4, *p* < 0.05) than that in the healthy controls. However, with respect to TrA, SWS in the lower abdominal wall (locations of 9 and 10) of DRA patients displayed higher value than that of the healthy controls (*p* = 0.007,* p* < 0.001, respectively).

**Table 3:** Comparison of shear wave speed of abdominal wall muscles at different locations

	Shear wave speed (m/s)		*p*
	DRA	Non-DRA	
Rectus abdominis (RA)			
Location 1	1.86 ± 0.24	2.00 ± 0.16	0.015
Location 2	1.79 ± 0.25	1.99 ± 0.18	0.001
Location 3	1.64 ± 0.22	1.90 ± 0.16	< 0.001
Location 4	1.65 ± 0.27	1.88 ± 0.20	0.001
Location 5	1.61 ± 0.19	1.62 ± 0.18	0.888
Location 6	1.58 ± 0.25	1.52 ± 0.20	0.341
External oblique (EO)			
Location 7	1.59 ± 0.14	1.80 ± 0.25	< 0.001
Location 8	1.59 ± 0.18	1.71 ± 0.15	0.009
Location 9	1.70 ± 0.21	1.86 ± 0.22	0.007
Location 10	1.70 ± 0.21	1.78 ± 0.19	0.190
Internal oblique (IO)			
Location 7	1.57 ± 0.15	1.58 ± 0.23	0.850
Location 8	1.65 ± 0.17	1.50 ± 0.20	0.002
Location 9	1.62 ± 0.18	1.57 ± 0.20	0.359
Location 10	1.63 ± 0.24	1.52 ± 0.20	0.068
Transversus abdominis (TrA)			
Location 7	1.59 ± 0.23	1.49 ± 0.24	0.112
Location 8	1.66 ± 0.24	1.35 ± 0.26	< 0.001
Location 9	1.71 ± 0.26	1.54 ± 0.19	0.007
Location 10	1.76 ± 0.30	1.44 ± 0.20	< 0.001

Data are presented as mean ± standard deviation values

The Pearson correlation analyses exhibited significant correlations between IRD and SWS in the 36 DRA patients (Table 4), specifically for the RA, IO, and TrA (*p* < 0.001, *p* < 0.001, *p* = 0.003, respectively). In contrast, no statistically significant correlation was found between SWS and muscle thickness (*p* > 0.05). In addition, the Pearson analysis between SWS and muscle thickness in the overall dataset (*n* = 60) revealed a significant correlation (*γ* = 0.238, 95%: [0.126, 0.360], *p* < 0.001).

**Table 4:** Correlation coefficients among the US variables in the 36 DRA patients

Findings	Coefficient	95%CI	*p* value
SWS (m/s) versus IRD			
RA	0.574	0.318, 0.772	< 0.001
EO	0.125	− 0.180, 0.390	0.233
IO	0.589	0.334, 0.772	< 0.001
TrA	0.453	0.227, 0.679	0.003
Muscle thickness versus IRD			
RA	− 0.076	− 0.387, 0.215	0.330
EO	− 0.287	− 0.617, 0.078	0.089
IO	0.135	− 0.121, 0.398	0.216
TrA	0.021	− 0.222, 0.313	0.451

*US* ultrasound, *SWS* shear wave speed, *IRD* inter-rectus distance, *RA* rectus abdominis, *EO* external oblique, *IO* internal oblique, *TrA* transversus abdominis

**Fig. 6:**
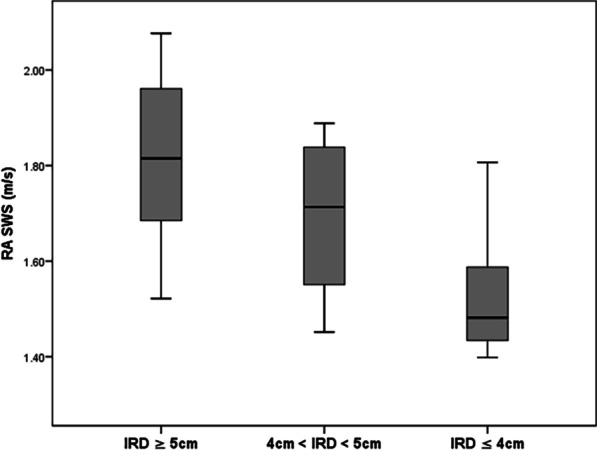
Boxplot of RA shear wave speed distribution in 36 DRA patients (*IRD* inter-rectus distance, *RA* rectus abdominis, *SWS* shear wave speed)

A total of 36 DRA patients were reclassified to 3 subgroups in terms of RA separation distance (subgroup1: IRD ≥ 5 cm, subgroup2: 4 cm < IRD < 5 cm, subgroup3: IRD ≤ 4 cm). Of note, the mean value of RA SWS in subgroup3 was significantly lower than that in the other two subgroups (subgroup3 vs. subgroup1, subgroup3 vs. subgroup2, *p* < 0.001, *p* = 0.011, respectively, Fig. 6). The larger the separation, the higher the shear wave speed in the RA. The mean SWS value of subgroup1 was 1.82 ± 0.17 m/s. No significant difference in SWS was found between subgroup1 and the control group (1.82 ± 0.17 m/s vs. 1.82 ± 0.13 m/s, *p* = 0.988).

**Fig. 7:**
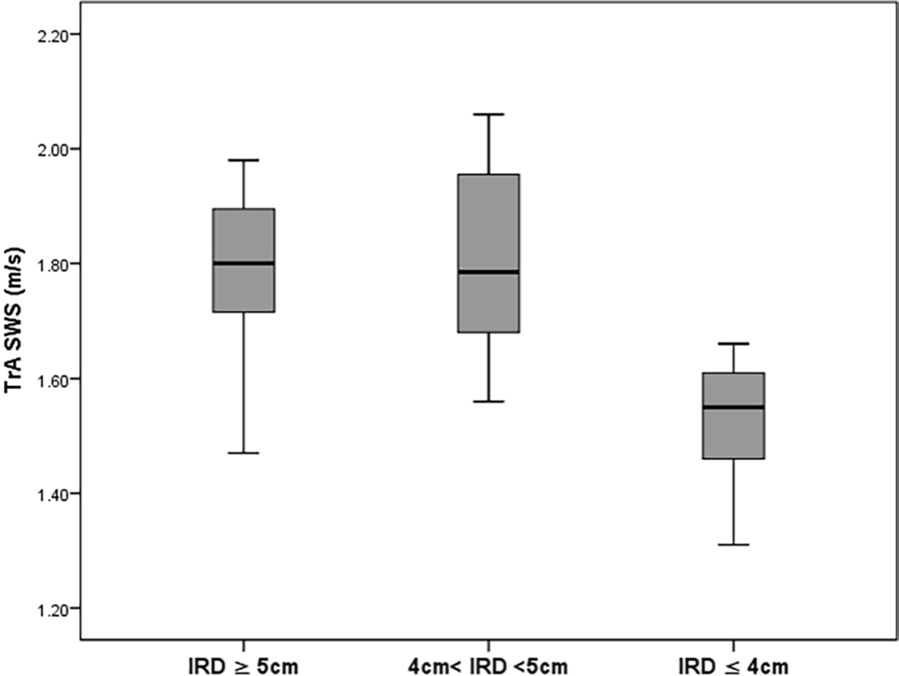
Boxplot of TrA shear wave speed distribution in 36 DRA patients (*IRD* inter-rectus distance, *TrA* transversus abdominis, *SWS* shear wave speed)

Similarly, the mean value of TrA SWS in subgroup3 was significantly lower than that in the other two subgroups (subgroup3 vs. subgroup1, subgroup3 vs. subgroup2, *p* < 0.001, *p* < 0.001, respectively, Fig. 7). No significant difference was found between subgroups 1 and 2 (*p* > 0.05).

## Discussion

Ultrasound is becoming more popular in the evaluation of different musculoskeletal abnormalities with excellent reproducibility [22]. With the recent developments in ultrasound elastography, it is now possible to quantitatively evaluate the stiffness of muscle [12–14]. There are several ultrasound elastography methods investigated around the world in the past twenty years, including strain elastography, acoustic radiation force impulse, shear wave elastography, and transient elastography [23]. As a semiquantitative approach, strain ratio values generated by strain elastography depend significantly on reference and target region of interest being above the same tissue, while not influenced by depth [24]. Shear wave speeds would probably decrease with increasing scanning depth [24]. Comparatively, more recent studies demonstrated that, in terms of inter- or intra-operator variability, shear wave elastography is a more reliable and widely used tool for quantitatively assessing muscle stiffness [25–29].

**Fig. 8:**
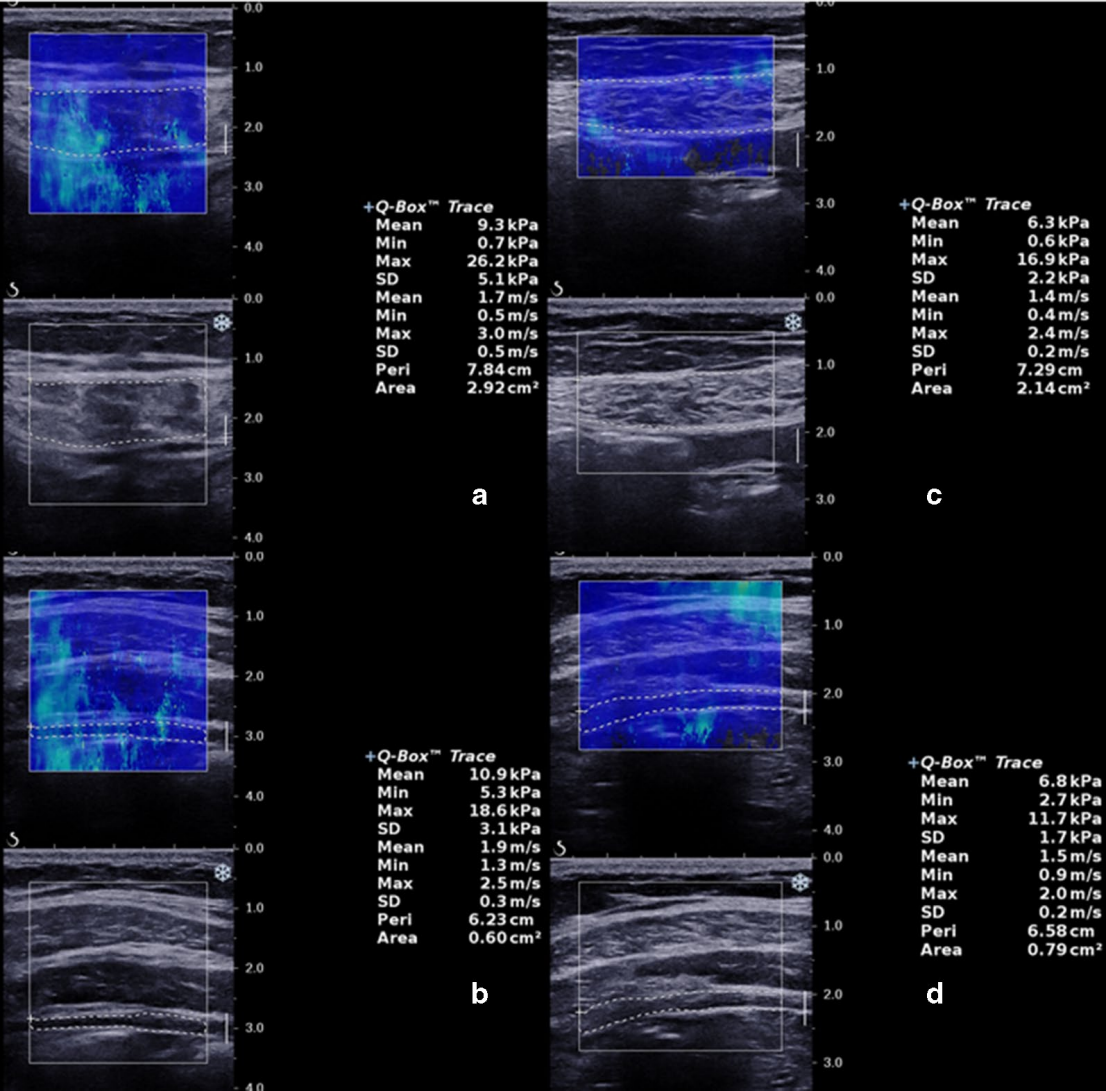
Shear wave elastography of patients with DRA. One forty-year-old patient with inter-rectus distance of 5.12 cm showed higher shear wave speed in RA and TrA (**a** 1.7 m/s, **b** 1.9 m/s). Comparatively, another thirty-two-year-old patient with inter-rectus distance of 3.36 cm exhibited lower shear wave speed in RA and TrA (**c** 1.4 m/s, **d** 1.5 m/s)

The main purpose of this study was to compare elasticity of abdominal wall musculature in individuals with and without DRA. Partially consistent with the first part of our hypothesis, we found a significantly lower SWS in the RA (*p* = 0.003) and a higher SWS in the TrA muscle (*p* < 0.001) in participants with DRA compared with the age-matched control group without previous pregnancy. However, the correlation between muscle elasticity and IRD should be interpreted with caution (Fig. 8). As SWS in both muscles were positively correlated with IRD (*p* < 0.05), the greater the IRD, the higher the SWS in RA. In addition, the value of SWS in EO unexpectedly decreased in DRA patients (1.65 ± 0.15 vs. 1.79 ± 0.14, *p* = 0.001). To the best of our knowledge, the present study is the first to reveal these findings about muscle elasticity in DRA patients based on the specific 10-location setting.

The reliability of IRD measurement in postpartum women using B-mode ultrasound has been widely validated by multiple studies in the past decade [7–9]. In response to our primary goal of muscle elasticity assessment, the ICC values obtained in the current study indicate moderate to high intra-operator reliability, especially for the RA and TrA muscles. This finding was in agreement with our previous study of SWE in incisional hernia patients [15]. However, it was noted that the ICC values for EO and IO were relatively lower, which might be attributed to the impact of patient breathing on superficial oblique muscles [30].

The maximum separation distance was located at the umbilicus, which is in line with the DRA classification system suggested by Rath et al. [19]. Contrary to Liaw’s finding, no inter-rectus separation was found among the 24 nulliparous healthy subjects, irrespective of the locations along the midline (M1–M5). Of note, the mean age of the control subjects differed between the two studies (31.9 ± 4. 1 vs. 26.7 ± 4.7 mm). However, both studies complied with the commonly accepted definition of DRA, with no more than 1 to 2 cm separation [3].

Our study is unique in the use of SWE in 10-location settings to better quantify the elasticity of abdominal muscles in an objective approach. Given the current evidence of morphological changes and abdominal muscle functional deficits present during pregnancy [8], a group of nulliparous healthy women was enrolled as controls in this study. Generally, the range of SWS values acquired in the four abdominal wall muscles (0.85–2.08 m/s) was similar to the values reported by others [15, 25–28]. From a biomechanical viewpoint, the abdominopelvic cavity is a cylinder enveloped by muscles, tendons, and bony structures. According to Pascal's principle [31], any pressure generated within the abdominopelvic cavity is transmitted equally to the walls of that cavity. In response to increased abdominal pressure, the muscular abdominal wall contracts to generate counter-pressure. If intra-abdominal pressure exceeds abdominal wall pressure, the abdominal wall will rupture at its weakest point, causing herniation. Correspondingly, the rectus abdominis in DRA patients, which is considered the margin of the weakest point, displayed a lower SWS value than that in healthy controls, especially for the locations above and at the umbilicus. This difference in muscle elasticity should be considered when making treatment plans that include a combination of physiotherapy and surgical repair [11]. However, subgroup analysis in 36 DRA patients showed a positive correlation between IRD and SWS. This can be explained by the biomechanical and pathological changes in muscle fibers as the inter-rectus separation increases [25–27]. The composition of the RA, including muscle fibers, connective tissue, and adipose tissue infiltration, might vary at different stages of DRA progression, resulting in changes in shear wave speed at the later stage.

Converse to our results in incisional hernia patients [15], the SWS of the EO muscle in DRA patients decreased in comparison with healthy subjects. This decrease mainly resulted from the biological and physical differences between the two studies. According to the law of Laplace [31], once a hernia has formed, it will continue to enlarge in size due to the increase in wall tension at that location. The wall tension is greatest at the point of the largest radius and the thinnest wall. Hence, it could be hypothesized that these muscles are more likely to be subjected to higher tension. In this study, no hernia was found among the 36 DRA patients. In addition, the positive correlation between SWS and muscle in the overall dataset (*γ* = 0.238, *p* < 0.001) may be explained by the significantly decreased EO muscle thickness (*p* < 0.001) with less volume of fibers in postpartum women that leads to slower shear wave propagation.

Similar to the EO muscle, the mean thickness of the TrA muscle decreased in DRA patients. Nonetheless, the tension of the TrA muscle increased. This finding is in agreement with the results of the anatomical study by Wingerden et al. [32], which demonstrated that the TrA muscle, as the deep abdominal wall muscle, played an important role in the etiology of DRA. Typical for pregnancy is the space requirement of the growing uterus, which increases intra-abdominal pressure. The muscular tissue adapts more rapidly and to a further extent than collagenous fascial sheets [33]. Hence, the posterior rectus fascia sheath, mainly formed by the aponeuroses of the TrA, is not expected to be as lax as the muscular dominant tissue. Comparison of the TrA SWS values among different locations also implicates the significance of the lower abdominal wall in preoperative treatment, including exercise the antenatal and postnatal periods.

Consistent with a prior study by Murillo et al. [34], the symptom of lumbopelvic pain was significantly increased in the DRA group (*p* < 0.001). The differences in shear wave speed between lumbopelvic pain and asymptomatic individuals can be attributed to the increase of connective tissue due to fibrotic proliferation.

The present study establishes a method for measuring the shear wave speed of the abdominal wall muscles in DRA patients. In agreement with other researchers [13, 14], shear wave elastography provides a direct estimation of muscle force. Since the management options of DRA vary and will depend not only on the degree of separation but the flaccidity of the anterior abdominal wall as well [35], accurate and objective assessment of muscle elasticity may potentially assist in the clinical management. For example, simple physical therapy might be effective for those who present mild to moderate diastasis with normal muscle elasticity. On the other side, surgical complications following rectus diastasis repair, such as infection, mesh extrusion, and recurrence, could be avoidable based on the comprehensive preoperative evaluation by shear wave elastography.

Nevertheless, imaging of only a few parts of the muscle is insufficient to generalize the results to the whole muscle. Furthermore, postprocessing steps are necessary for more representative results. Moreover, we only recruited limited patients and nulliparous healthy controls in our study. Thus, whether these results apply to postpartum subjects without DRA is unclear. Further research is warranted.

To summarize the main findings of our study, the application of SWE for assessing abdominal wall muscles is feasible and credible in DRA patients. Our study revealed lower SWS in the RA and higher SWS in the TrA. Meanwhile, SWS positively correlated with IRD in the two muscles (RA and TrA, respectively). Thus, further caution should be paid to the interpretation of the correlation between SWS value and IRD in the RA.

## Data Availability

The datasets used and/or analysed during the current study are available from the corresponding author on reasonable request.

## Abbreviations

DRA: Diastasis recti abdominis
EHS: European Hernia Society
EO: External oblique muscle
ICC: Intra-class correlation coefficient
IO: Internal oblique muscle
IRD: Inter-rectus distance
RA: Rectus abdominis
SWE: Shear wave elastography
SWS: Shear wave speed
TrA: Transversus abdominis
US: Ultrasound
